# The Impact of Water as an Additive on the Elution of Some Basic Organic Compounds in Supercritical Fluid Chromatography

**DOI:** 10.3390/molecules29092124

**Published:** 2024-05-03

**Authors:** Muhamad Yahia Kazmouz, Attila Felinger

**Affiliations:** 1 Department of Analytical and Environmental Chemistry and Szentágothai Research Center, Ifjúság útja 6, H-7624 Pécs, Hungary; 7chemaster@gmail.com; 2 HUN-RES-PTE Molecular Interactions in Separation Science Research Group, Ifjúság útja 6, H-7624 Pécs, Hungary; 3 Institute of Bioanalysis, Medical School, University of Pécs, Szigeti út 12, H-7624 Pécs, Hungary

**Keywords:** supercritical fluid chromatography, additive role, water as additive, modifier, basic analytes

## Abstract

In this study, water was used as an additive in the methanol-modified carbon dioxide-based eluent for the elution of some basic organic compounds from a hybrid silica column via supercritical fluid chromatography (SFC). The experiments were applied to sulfonamides, propranolol, and other organic nitrogen compounds involving aromatic rings from different classes of amine, pyrimidine, and purine with different pKa values (the pKa values for the studied analytes range from 4.6 to 10.4). The results revealed different responses to the different percentages of water addition. Adding 1~2% of water to the modifier (methanol) led to a positive effect manifested by more symmetrical peak shapes and reduced retention times for most compounds. The key factor for this improvement in the properties of chromatographic peaks is due to the adsorption of water on the silanol groups of the stationary phase, consequently resembling the phenomena observed in hydrophilic interaction liquid chromatography (HILIC). Moreover, the availability of hydrogen bond acceptor and donor sites in the analyte structure is an important factor to be considered when adding water as an additive to the modifier for improving the chromatographic peaks. However, introducing water in an amount higher than 3% resulted in perturbed chromatographic signals. It was also found that water as an additive alone could not successfully elute propranolol from the hybrid silica column with an acceptable peak shape; thus, the addition of a strong base such as amine salts was also necessary. The proposed use of a particular amount of water in the mobile phase could have a positive effect compared to the same mobile phase without water, improving the chromatographic peak properties of the elution of some basic organic compounds from the hybrid silica column.

## 1. Introduction

Quality control in chemical industries simply requires facilitating analytical methods that save both time and chemicals. In this regard, supercritical fluid chromatography (SFC) is considered complementary to other analytical techniques such as liquid chromatography (LC) thanks to the short analysis time and low consumption of organic solvents. One of the most attractive practical aspects of SFC is that it can be tuned to analyze both hydrophobic compounds in the nonpolar phase [1] and hydrophilic compounds in the polar phase [2].

It has been known for years that incorporating supercritical CO_2_ as a mobile phase in SFC has limited use due to its low polarity, which is very close to liquid pentane or liquid hexane [3]; therefore, the majority of SFC experiments are set up with alcohol modifiers to improve the chromatographic results of the separation [4].

Adding small amounts (0.5–1%) of very polar compounds (called additives) to the mobile phase is highly favorable for expanding the range of the polarity of the analytes that can be analyzed with SFC [5]. For instance, the separation of basic analytes (e.g., nitrogen-containing molecules) is considered problematic, as reported by Berger et al. [6], who studied the elution of a dibenzylamine and benzylamine mixture using different stationary phases. According to their results, the elution using methanol-modified CO_2_ did not result in good peak shapes. However, in the presence of isopropylamine (IPAm) as an additive, an improvement in the peak shapes was obtained. Similar behavior was observed in the elution of benzene dicarboxylic acid (polyfunctional carboxylic acids) from two diol and sulfonic acid columns using methanol–carbon dioxide mixtures. The peak shapes were poor, and long retention times were obtained, but incorporating very polar additives (0.5% of either citric acid or trifluoroacetic acid) substantially improved the peak shapes [7].

Generally, in SFC, the apparent pH of CO_2_ modified with alcohols is around 5, which is a result of alkyl carbonic acid formed from the reaction between CO_2_ and alcohol. Carbonic acid may also be formed, however, in the presence of water as an additive and CO_2_ [8,9]. Thus, the basic compounds surrounded by this acidic environment will undergo an ionization process, and protonated molecules will be formed, causing unfavorable interactions with the strong adsorption sites on the stationary phase. For this reason, the use of water as one of the mobile phase components supposedly helps to cover the strong adsorption sites such as the silanol groups; consequently, the retention mechanism would be the same as in HILIC, which is basically described as the partitioning of the analytes between the bulk mobile phase and a water-rich layer of mobile phase adsorbed on the stationary phase. This mechanism is consistent with the outcomes of earlier studies [10,11,12,13], which emphasized that the mobile phase components (i.e., water molecules) adsorb on the stationary phase, thus modifying the surface chemistry of the packing material.

In related studies that focused on improving the efficiency and peak shapes of the studied compounds, uracil was eluted with good peak shapes from the polar stationary phases (silica-bonded diol, cyanopropyl, and 2-ethyl pyridine) without the use of any additives in the mobile phase [12]. For sulfonamides, SFC is considered a greener and faster alternative than HPLC, which is a suitable choice for the quantitative analysis of these compounds. Perkins et al. showed that a gradient mode of a simple MeOH-CO_2_ mobile phase was able to separate a mixture of eight sulfonamides from a packed amino-bonded Spherisorb column [14]. Afterward, Combs et al. separated eight sulfonamides from silica and an amino column connected in series using a gradient MeOH-CO_2_ mobile phase to obtain a good resolution without peak co-elution [15]. In another study, nine components of sulfonamides were baseline-separated within 4 min in isocratic elution mode using a 1.8 µm RX-SIL (fully porous silica) column [16].

There is a lack of studies that report the enantiomeric separation of racemic propranolol hydrochloride salt from a chiral stationary phase using the MeOH-CO_2_ mobile phase without additives [17], while different attempts are available for propranolol HCl separation from the non-chiral columns without additives. Bailey et al. showed that propranolol HCl eluted with a tailed peak from both aminopropyl and diol-bonded silica columns using the MeOH-CO_2_ mobile phase, but for better chromatographic results, the use of triethylamine (TEA) as an additive (to reduce the interactions between solute and free silanol groups on the surface of the stationary phase) was recommended to obtain a symmetrical peak [18]. Other stationary phases, e.g., Luna HILIC Diol, were tested for the same purpose, but the best result with a baseline separation for a mixture containing propranolol HCl was obtained on a Princeton SFC 2-EP column in a gradient elution mode of the MeOH-CO_2_ mobile phase without the use of additives [19]. More information about the propranolol HCl elution from different stationary phases with or without additives can be found in [20].

### 1.1. Additive Behavior in SFC

The influence of additives in SFC has been studied by several research groups [19]. Employing additives was introduced as an approach for treating the associated problems with the peak elution of basic analytes [21,22], such as improving the elution of phenylthiohydantoin amino acid (PTH-AA) derivatives using supercritical CO_2_ modified with methanol containing tetramethylammonium hydroxide as an ion-pairing reagent. A study [23] indicated that the use of ion-pairing agents as additives for enantiomeric compound separation improves separation efficiency. The results of another study [24] revealed that the peak shapes and retention times of organic nitrogen-containing compounds were significantly improved with the modified CO_2_-containing isopropylamine additive compared to those without the use of additives.

Regarding the role of additives in improving the peak shape and separation efficiency, different mechanisms have been suggested. These include (i) covering the residual silanol groups of a bonded silica stationary phase, as silanols are one of the main reasons for the poor quality of peak shapes [25]; (ii) ion pair formation between the ionizable solutes and additives [26]; (iii) the suppression of solute ionization (for instance, a strong acid additive could help ion suppression for less acidic solutes [5], and the same concept can be applied for basic solutes using a strong base additive); (iv) additive adsorption on stationary phases may enhance or cancel specific interactions between analytes and adsorbed additives [10], and another result of additive adsorption is that it can modify the polarity of a stationary phase [11]; and (v) modifying the apparent pH of the mobile phase, which can lead to different ionization states of the analytes, which in turn, influences the retention of the analytes [8].

### 1.2. Using Water as an Additive in SFC

The main features of water—besides being the most environmentally friendly solvent—are that it has a very high polarity character (i.e., solvent polarity parameter *Ρ′* = 10.2 for water, *Ρ′* = 5.1 for methanol, and *Ρ′* = 0 for pentane). Water can act both as a hydrogen acceptor and a hydrogen donor. These properties are expected to give water a significant role as an additive with CO_2_-based mobile phases in SFC [27].

Recent studies about introducing water to the modified CO_2_ [10] have shown that water competes with methanol to adsorb on the hybrid silica stationary phase, which is considered one of the main reasons influencing the retention of the compounds that can form hydrogen bonding interactions.

An interesting study was conducted by Roy et al., where they measured the polarity of a mixture of CO_2_ modified with 10–40% methanol with and without adding water, and they observed that the use of 40% methanol as a modifier containing 6% water resulted in very little change in the polarity of the mixture compared to the mixture without water. The results were obtained by measuring the transition energy of Nile Red as a solvatochromic probe [28]. A similar experiment was performed by West et al., referring to a very slight change in the mobile phase polarity when the methanol content in the eluent was 60%, containing 1.2% water in the total mobile phase [8]. Considering these findings and, at the same time, the ability of water as an additive in the ternary mobile phase to impart an influence on the separation process, we found that the influence of water on the analyte’s retention is attributed to water adsorption on the stationary phase, consequently modifying the interaction between solutes and the stationary phase.

The use of water as an additive in SFC has been utilized by a few research groups. For instance, it was observed that the separation efficiency increased for compounds that have a higher retention factor than water on a silica-bounded cyclofructan-6 (HILIC) stationary phase; additionally, the gain in efficiency was lucid for most of the analytes on the bare silica stationary phase [29]. Lafosse et al. used CO_2_ modified with 21.6% of a methanol–water–triethylamine mixture for phospholipid separation on a Zorbax silica column [30]. Also, water was used with methanol-modified CO_2_ to elute monosaccharides and polyols from silica and trimethylsilyl (TMS)-bonded silica stationary phases in SFC [31]. Ashraf-Khorassani et al. reported that the presence of water in the mobile phase yielded sharper peaks and enhanced the separation resolution for the sulfonamide mixture and the neutral compounds’ mixture (containing caffeine and uracil analytes) from a bare silica column, but for the mixture of acidic compounds, similar chromatographic data were obtained with or without water [13].

According to the results of [12], it was found that water was significantly more useful and superior to formic acid to elute water-soluble nucleobases from polar stationary phases with sharp peaks using a gradient mode of alcohol-modified CO_2_.

The use of a combination of water and another compound, such as ammonium hydroxide, as an additive in the mobile phase [32] gave rise to enhanced peak shape and selectivity of polar compounds because of the formation of a chaotropic agent (carbonate/bicarbonate anion), which obstructs undesired hydrogen bonds between the analytes and the stationary phase. Regarding the effect of water usage on chiral separation, the results demonstrated that a dramatic gain in the separation efficiency of hydrophilic phases—compared to hydrophobic phases—also leads to a decrease in the retention times of hydrophilic stationary phases [28]. Other applications of using water as an additive are reported in [33,34].

In the present work, water was evaluated as an additive to a methanol-modified CO_2_-based eluent for the elution of some basic analytes from a hybrid silica column. The evaluation was performed based on a comparison between the chromatographic results obtained with the modified mobile phase in the presence of water and without adding water. This attempt is a step toward operating SFC with less consumption of organic chemicals.

## 2. Results and Discussion

The use of water in the mobile phase was required for our experiments, so it is worth noting that the water content in the mobile phase is limited because of the miscibility issue between water and CO_2_, which can be enhanced by adding higher amounts of the polar solvents in the total mobile phase volume [35]. In our study, 7% water in the modifier showed a disturbed baseline even when a small portion of the modifier (3%) was in the mobile phase; therefore, 6% water in the modifier was the maximum percentage that was used in this study.

**Figure 1:**
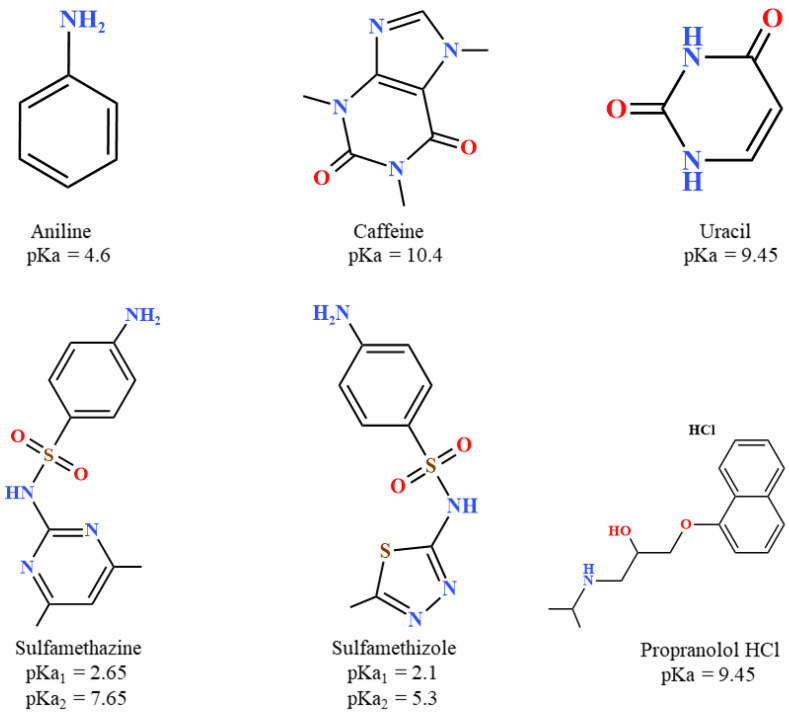
Molecular structures of the polar analytes with their pKa values.

The analytes used in this study are shown in Figure 1. All of these compounds or their derivatives are widely used in the pharmaceutical industry. They were chosen to exhibit a distinctive behavior on the Viridis BEH column, as will be shown in the next sections.

### 2.1. Peak Shapes and Symmetry Factor

**Figure 2:**
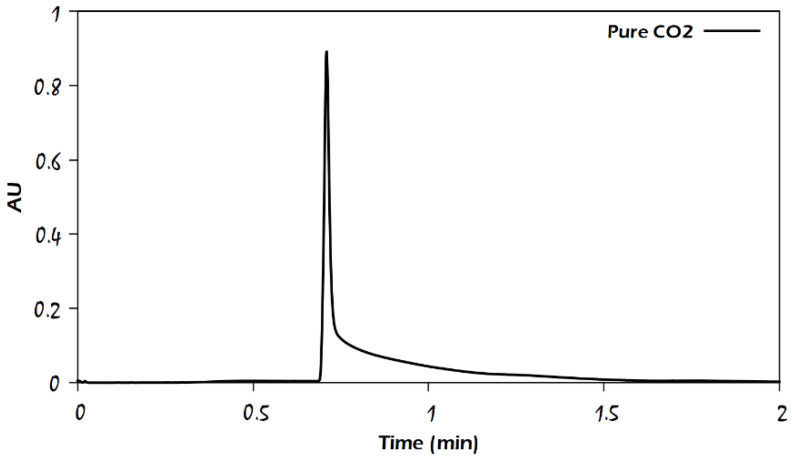
Chromatogram of aniline on the Viridis BEH column (50 × 3 mm) using 100% CO_2_ as mobile phase at 26 °C column temperature, back pressure 150 bar, flow rate of 2 mL/min, and UV detection at 204 nm.

**Figure 3:**
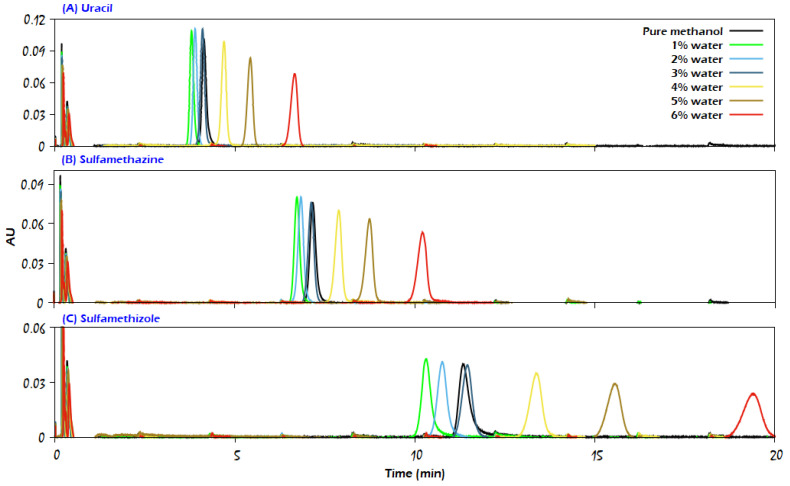
Influence of different water content on the elution of (**A**) uracil, (**B**) sulfamethazine, and (**C**) sulfamethizole on the Viridis BEH column with CO_2_/modifier (97:3, *v*/*v*). The modifier was either neat methanol or methanol with the addition of water in different percentages, as indicated in the figure. Other analytical conditions are the same as in Figure 2.

The preliminary experiments on the Viridis BEH (hybrid silica) column were performed using neat supercritical CO_2_ as the mobile phase. As expected, CO_2_ was not able to elute any of the analytes except aniline, for which a tailing peak was obtained, as shown in Figure 2. This peak eluted early (approximately three times the void time). The short retention time may be caused by the low probability of aniline forming a hydrogen bond with the adsorption sites of the stationary phase because the only lone pair on the nitrogen atom in the aniline molecule interacts with pi electrons in the benzene ring; thus, it can be easily eluted with CO_2_. On the other hand, the role of water as an additive in the mobile phase on the retention of analytes could be more obvious with other analytes. As shown in the comparison of chromatograms in Figure 3, by adding 1–2% water as an additive to the modifier (methanol) that forms 3% of the mobile phase, the peak shapes somewhat improved for the analytes uracil, sulfamethazine, and sulfamethizole.

On the one hand, water was able to interact with the adsorption sites (silanol groups) on the stationary phase, which have a high affinity for polar molecules; thus, it reduced the interaction possibility between the solutes and these sites. On the other hand, by adsorbing water on the stationary phase, the polar analytes eluted from the column because of partitioning into this layer and the mobile phase, but an excess of water in the mobile phase presented the possibility for an excess amount to surround the polar molecules; thus, they retained in the solvated stationary phase with water for a longer time, resulting in wider peaks and less efficiency.

**Figure 4:**
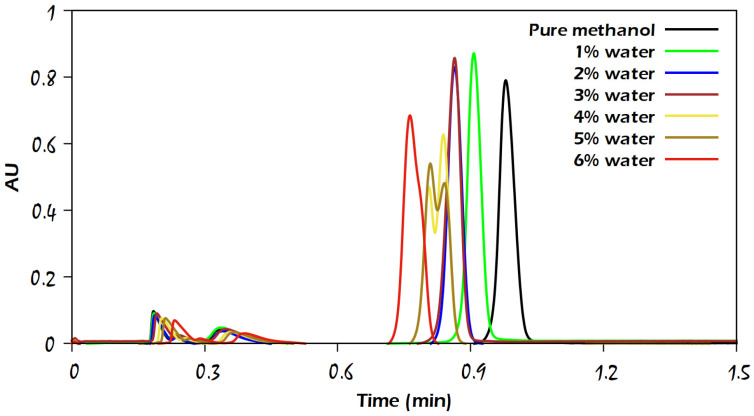
Comparison of chromatograms for the influence of different water percentages on the elution of caffeine with CO_2_/modifier (97:3, *v*/*v*). The modifier was either neat methanol or methanol with the addition of water in different percentages, as indicated in the figure. Other analytical conditions are the same as in Figure 2.

For the caffeine solute, as represented by the chromatograms in Figure 4, adding water up to 3% to the modifier resulted in very little improvement in the peak shapes and slightly decreased retention time, similar to the elution with methanol-modified CO_2_ (without water). The reason that adding water to the mobile phase could not greatly contribute to the enhancement of caffeine retention may be due to the absence of the hydrogen bond donors, that only three acceptors were available, and because of the weakly hydrated flat faces of the caffeine molecule. Furthermore, adding more than 3% of water to the modifier gave rise to deformed peaks for caffeine. This caused unusual results of the symmetry factor and efficiency, as will be discussed in a subsequent section.

The elution of a racemic mixture of (±) propranolol HCl on a hybrid silica column in the presence of water as an additive with methanol-modified CO_2_ was not successful in eluting the solute with an acceptable peak shape. Therefore, mixing a small amount of a basic additive (for ion suppression) with the modifier was required to obtain a convenient retention time with a good peak shape.

**Figure 5:**
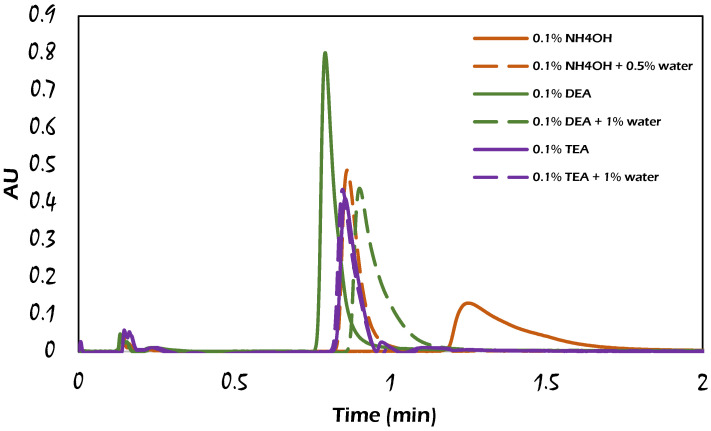
Comparison of chromatograms for the influence of different mobile phase additives on the elution of (±) propranolol HCl with the mobile phase composition: CO_2_/modifier (85:15, *v*/*v*), dashed lines: additive with water; solid lines: additive without water. The rest of the analytical conditions are the same as in Figure 2.

The effects of three common basic additives, namely diethylamine (DEA), ammonium hydroxide (NH_4_OH), and trimethylamine (TEA) with or without water, were studied for propranolol HCl elution. The mixture of an additive and modifier formed 15% of the total mobile phase. The results obtained for the different basic additives employed in the modifier were clearly different from each other, as presented in Figure 5.

The combined additive of 0.5% water and 0.1% ammonium hydroxide solution was added to the methanol modifier, which positively influenced the peak shape, signal intensity, and retention time in comparison to the addition of 0.1% ammonium hydroxide but no water. This combination of additives was reported as a chaotropic agent by Liu and co-workers [32] (see Section 1.2). Incorporating an amine salt with water as an additive caused different effects on the propranolol HCl elution from the column. As noticed, adding 1% water to methanol containing 0.1% TEA did not affect the propranolol HCl elution compared to that without water, while with the use of DEA instead of TEA, at the same concentration, the presence of water caused a decrease in the signal intensity of the propranolol HCl peak to half, and the peak shape was negatively influenced compared to the use of DEA without water, probably due to the slow mass transfer of the analyte in the presence of adsorbed water on the stationary phase. The aforementioned observations with propranolol HCl separation emphasize that water played propitious and unpropitious roles in the elution process according to the mobile phase components.

**Figure 6:**
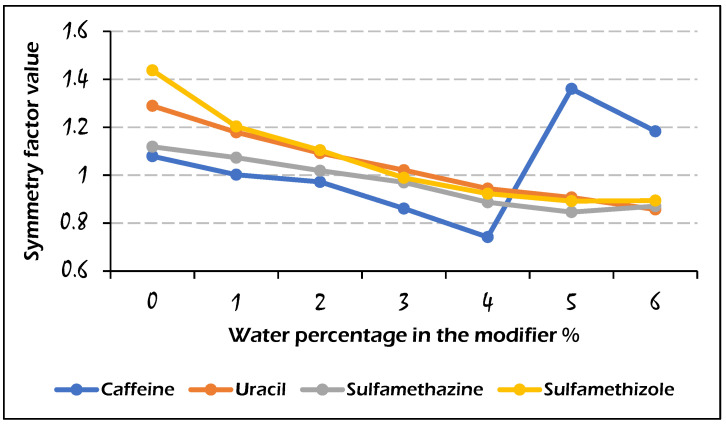
Effect of the water percentage on the symmetry factor values for the elution of four analytes on the Viridis BEH column with the mobile phase composition: CO_2_/modifier (97:3, *v*/*v*).

The symmetry factor of the recorded peaks was estimated as an indicator for the peak quality of caffeine, uracil, sulfamethazine, and sulfamethizole, which are shown in Figure 3 and Figure 4, in accordance with the water amount in the modifier. The corresponding values for the symmetry factor of each analyte were very similar to those for the other studied compounds (except for caffeine, where there was an anomalous trend when the water amount was higher than 3%). The results in Figure 6 show that the symmetry of peaks was improved (symmetry factor value close to 1) by adding water to the modifier, indicating that 2–3% water in the modifier was enough to treat the peak tailing for the analytes studied.

### 2.2. Retention Time and Peak Efficiency

According to Figure 4, caffeine was easy to elute from the column using the modified CO_2_ with methanol in the presence of water or even without water; moreover, we noticed that caffeine eluted earlier when the water percentage in the modifier increased, which can be attributed, as previously mentioned, to the relatively low hydrophilic character of caffeine.

As can be seen in Figure 3, with 1% water content in the modifier, the retention times of uracil, sulfamethazine, and sulfamethizole decreased. Then, as the water content increased in the modifier, their retention time increased accordingly. This might be the result of more analyte molecules being surrounded by the water molecules, thus increasing the analyte attraction toward the adsorbed water molecules on the stationary phase. This can also be the reason why the high water content has a negative impact on the symmetry factor of the peak shape of these compounds, as also discussed earlier in Section 2.1. As also noticed from the chromatograms in Figure 3, the signal intensity of the peaks first increased when using 1% of water in the modifier, and then, as the water percentage increased, the peak intensities became smaller because of the increased retention times.

As shown in Figure 3, sulfamethizole was the most retained compound among the analytes with or without water addition, which may be due to the large number of hydrogen bond donors and acceptors present in the sulfamethizole molecule, enabling it to retain longer in the stationary phase.

The effect of water on the propranolol HCl retention times can be either compatible or incompatible, as shown in the chromatograms of propranolol HCl in Figure 5. Adding 1% water to the modifier in the presence of the DEA additive resulted in a negative impact, where a higher retention and a wider peak shape were obtained. This was presumably due to the formation of a positively charged amine in the DEA molecule (note that the lone pair of electrons on the nitrogen atom can accept a proton from water, resulting in positively charged nitrogen), causing less efficiency for suppressing the ionization of basic propranolol molecules, producing a tailing peak and higher retention time than the peak resulting from the use of DEA additive without water addition.

Incorporating water with TEA as an additive (where TEA has less basicity than DEA because of the steric hindrance of the TEA molecule) yielded an additive mixture that did not affect the propranolol HCl retention time compared to the case when TEA was used alone. Meanwhile, the use of ammonium hydroxide solution alone could not provide total ion suppression of propranolol HCl because the pKa***_(NH4OH)_*** (9.26) is slightly smaller than pKa***_(propranolol)_*** (9.45), accordingly, a tailed propranolol HCl peak was obtained. However, in the case of adding a mixture of water and ammonia solution as an additive to the mobile phase, it was able to form the carbonate/bicarbonate anion under SFC conditions (as mentioned earlier in Section 2.1), which is responsible for the improvement in the chromatographic results of the hydrophilic analytes. In this regard, the use of DEA alone as an additive in the modifier gave the best result for the propranolol HCl elution. Thus, an important conclusion can be emphasized here that water can have a significant effect on retention by interacting not only with components in the stationary phase but also with other components of the mobile phase.

**Figure 7:**
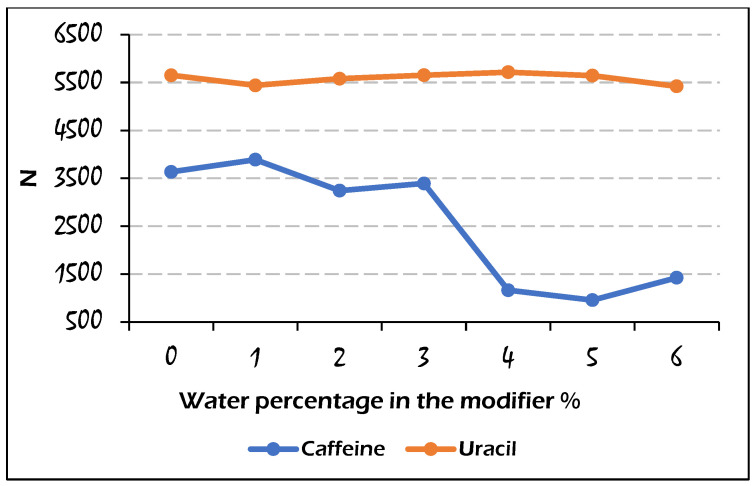
Effect of the water percentage on the calculated efficiency values for caffeine and uracil elution on the Viridis BEH column with the mobile phase composition: CO_2_/modifier (97:3, *v*/*v*).

**Figure 8:**
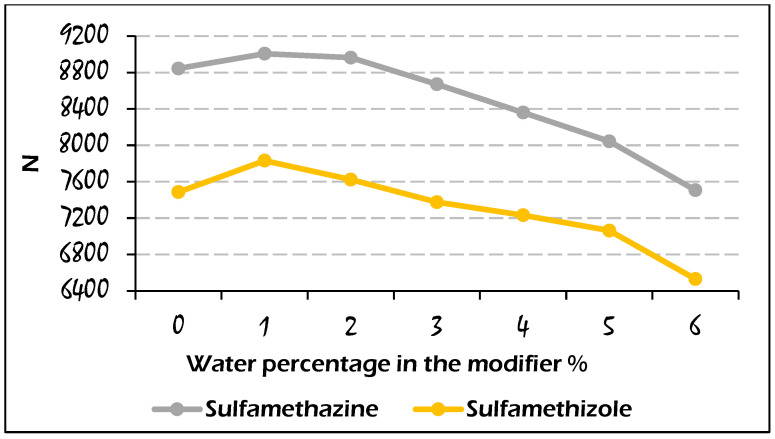
Effect of water percentage on the calculated efficiency values for the elution of sulfamethazine and sulfamethizole on the Viridis BEH column with the mobile phase composition: CO_2_/modifier (97:3, *v*/*v*).

The efficiency of the analyte peaks was evaluated by plotting the amount of water against efficiency, as represented in Figure 7 and Figure 8. Admittedly, since we observed different trends in water use for the elution of the studied analytes, it was expected that the efficiency values for those analytes would exhibit different trends as well. For instance, the efficiency of the uracil peak was not influenced by the water amount under the used range of water content, but for the caffeine peaks with a water amount higher than 3%, a loss in efficiency was observed, which was caused by the disruption of chromatographic peaks. In comparison, for sulfamethazine and sulfamethizole analytes, a slight increase in the plate counts at 1% of water was observed, followed by a decreasing trend due to the wide peak shapes. Also, it is obvious that the difference in efficiency (*N* values) between two consecutive points of sulfamethizole was larger than those for the other analytes. This means that the presence of water could largely affect the peak efficiency of sulfamethizole more than the rest of the analytes, which might be attributed to the high number of hydrogen bond acceptor/donor sites (7/2) in the molecule of sulfamethizole.

### 2.3. Effect of the Modifier Percentage

It is widely known in SFC that increasing the polar modifier proportion in the mobile phase, even in a small amount, increases the eluent strength, thereby increasing the analyte–mobile phase interactions and, consequently, modifying the retention of solutes [8]. In our study, two proportions of the modifier (3% and 5%) were used in the mobile phase to investigate the impact of increasing the amount of modifier containing water as an additive on the elution of uracil, sulfamethazine, and caffeine analytes. The water content in the modifier was between 0 and 6%.

**Figure 9:**
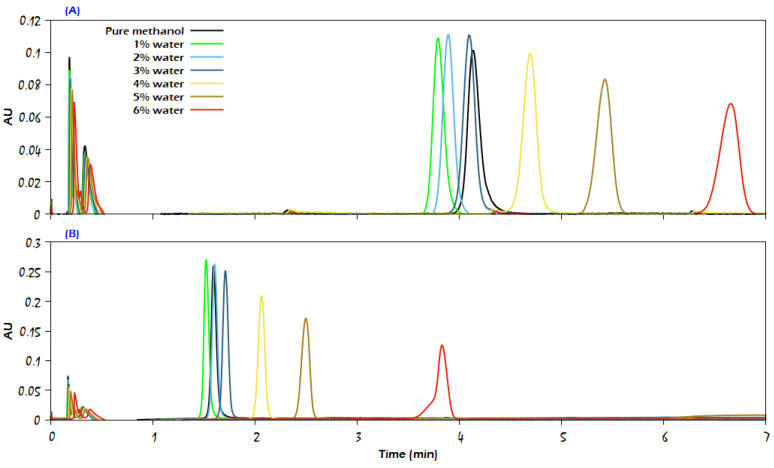
Comparison of the influence of increasing modifier percentage on the elution of uracil on the Viridis BEH column, where the mobile phase consisted of (**A**) CO_2_/modifier (97:3, *v*/*v*) and (**B**) CO_2_/modifier (95:5, *v*/*v*). The modifier was either neat methanol or methanol with the addition of water in different percentages, as indicated in the figure. Other analytical conditions are the same as in Figure 2.

**Figure 10:**
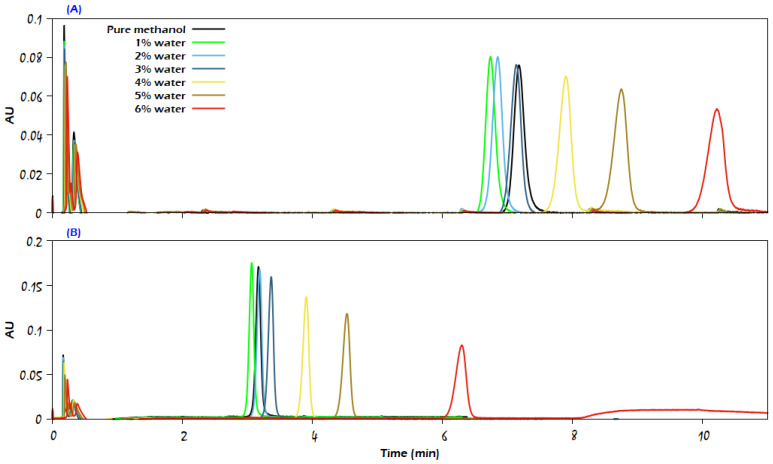
Comparison of the influence of increasing modifier percentage on the elution of sulfamethazine from the Viridis BEH column: (**A**) CO_2_/methanol (97:3, *v*/*v*); (**B**) CO_2_/methanol (95:5, *v*/*v*). The modifier was either neat methanol or methanol with the addition of water in different percentages, as indicated in the figure. The rest of the analytical conditions are the same as in Figure 2.

**Figure 11:**
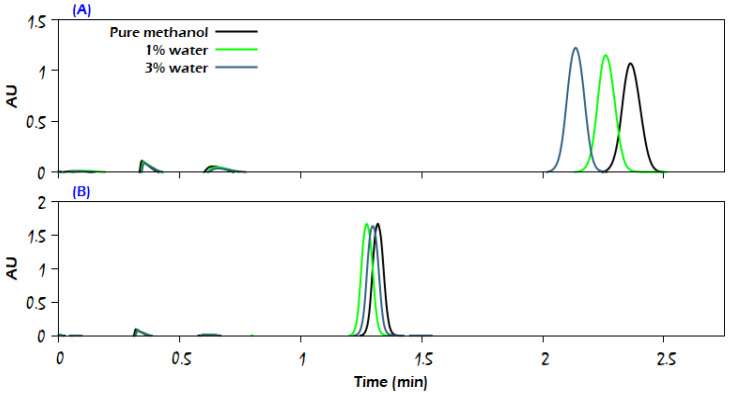
Comparison of the influence of increasing modifier percentage on the elution of caffeine with a flow rate of 1 mL/min: (**A**) CO_2_/modifier (97:3, *v*/*v*); (**B**) CO_2_/modifier (95:5, *v*/*v*). The modifier was either neat methanol or methanol with the addition of water in different percentages, as indicated in the figure. The rest of the analytical conditions are the same as in Figure 2.

The presented chromatograms in Figure 9, Figure 10 and Figure 11 of uracil, sulfamethazine, and caffeine, respectively, confirm that the use of a higher amount of the modifier significantly reduces the analyte retention while producing a sharp peak and more than doubling the intensity of the detector signal (the chromatograms of sulfamethizole at 95% CO_2_ + 5% modifier have the same trend of response as the chromatograms for both uracil and sulfamethazine; thus, they are not presented here). At the same time, a deformation in the analyte peak shape can be noticed due to the competition between the analyte and methanol to adsorb on the stationary phase, such as in the case of uracil at CO_2_/modifier (95:5, *v*/*v*), where the modifier was methanol containing 6% water. It is noteworthy that increasing the modifier content in the mobile phase apparently diminished the improvement role of water for the analytes; thus, the effective role of water in improving the properties of chromatographic peaks was more obvious at low modifier content in the mobile phase. For the sake of supporting the latter conclusion, additional experiments were conducted with CO_2_/modifier (90:10, *v*/*v*). The findings showed that the water content in the mobile phase had no effect on the retention time or peak symmetry of the compounds under investigation. Another set of measurements was carried out with CO_2_/modifier (99:1, *v*/*v*), but the eluent strength was not enough to elute uracil, sulfamethazine, and sulfamethizole, and only a single chromatographic peak was recorded for caffeine.

## 3. Experimental Procedures

### 3.1. Equipment

The measurements were acquired using an ACQUITY UPC^2^ system from Waters Corporation (Milford, MA, USA). The system is supplied with Empower 3 software for controlling the measurements and recording chromatograms. The instrument includes a binary pump, namely (1) a cooled pump for carbon dioxide and (2) a modifier pump that can be utilized up to four channels with integrated vacuum degassing; an auto-sampler with a 10 μL sample loop; a temperature-controlled column compartment for up to three columns; a regulator (convergence manager unit) to monitor and regulate the pressure of carbon dioxide and maintain the set back-pressure value; and a diode array U*V*/*V*IS detector. In this study, a hybrid silica Viridis BEH column 50 × 3 mm packed with 1.7 µm spherical particle from Waters Corporation (Milford, MA, USA) was employed, with the specific surface area of *S*_BET_ =340 m^2^/g and 130 Å pore size.

### 3.2. Standards and Chemicals

CO_2_ with a purity of 99.5% was used as the main mobile phase from Linde Group (Répcelak, Hungary). Methanol used as a modifier and water as an additive with HPLC grade purity were purchased from Fisher Chemicals (Loughborough, UK). The analytical standards with at least 99% purity were aniline (ANI), uracil (URA), (±), propranolol HCl (PRO), sulfamethazine (ZIN), and sulfamethizole (ZOL) from Sigma-Aldrich (Steinheim, Germany) and anhydrous caffeine (CAF) from Fluka (Seelze, Germany). Chemicals used in the experiments, such as diethylamine (DEA) and triethylamine (TEA), were obtained from Honeywell-Fluka (Seelze, Germany), and 32% ammonia solution was acquired from VWR (Fontenay-sous-Bois, France).

### 3.3. Chromatographic Experiments

The experiments were carried out with values set at 26 °C, 150 bar, 2 mL/min of column temperature, back pressure, and flow rate, respectively; the detector signal was recorded at 204 nm. All the samples were dissolved in methanol at a concentration of 0.5 mg/1.5 mL, except for aniline, for which the concentration was 1 µL/1.5 mL. The sample injection volume was 1 µL.

The symmetry factors for the chromatographic peaks were calculated with Empower 3 software using the following formula:

$$A_{s}=\frac{w_{0.05}}{2d}$$

where $w_{0.05}$ is the width of the peak at 5% of the peak height, and d is the distance between the perpendicular dropped from the peak maximum and the leading edge of the peak at 5% of the peak height. The separation efficiency was estimated with Empower 3 software according to the formula of the European Pharmacopoeia as follows:

$$N=5.54{\left(\frac{t_{R}}{w_{h}}\right)}^{2}$$

where $t_{R}$ is the retention time of the peak corresponding to the component; $w_{h}$ is the width of the peak at half-height.

## 4. Conclusions

This work is a complementary study to our previous work about the competitive adsorption of methanol and water on the applied hybrid silica column [1], which is an attempt to use SFC with less organic solvent consumption. The results indicate that employing a very small amount of water 0.03–0.06% in the total mobile phase can improve efficiency, decrease retention time, and produce more symmetrical peaks for the studied solutes, except for propranolol HCl. This improvement is assumed to depend on the availability of hydrogen bonding acceptor/donor sites in the analyte structure (as seen in uracil, sulfamethazine, and sulfamethizole), whereas in the case of the weak hydrogen bond acceptor analyte (caffeine), water could not significantly affect retention and efficiency. It is worth mentioning that, since there was no change in the polarity of the solvatochromic probe by adding water to the methanol–CO_2_ mixture (as mentioned in Section 1.2), it was found that the adsorption of a small amount of water on the stationary phase led to a change in the retention process of the analytes.

It was noticed that while the use of the modifier at a higher percentage in the mobile phase could positively impact the peak shape and intensity of the signal, and it also decreased the retention time significantly, it could diminish the role of water in improving the properties of chromatographic peaks. Meanwhile, it was observed that the use of water as an additive was not able to elute a strong basic solute such as propranolol HCl with an acceptable peak shape. Furthermore, it was observed that incorporating water as the additive could affect retention by interacting not only with components in the stationary phase but also with other components of the mobile phase, which was observed with the combined additive of H_2_O + NH_4_OH, which positively influenced the peak shape, signal intensity, and retention time in comparison to the addition of NH_4_OH alone. By contrast, the combined additive of diethylamine and water decreased the peak intensity, and a wider peak was obtained compared to the case of diethylamine alone. In summary, incorporating a limited amount of water as an additive to the mobile phase has a favorable role in improving the efficiency and peak shapes for the elution of some basic solutes from a hybrid silica stationary phase.

## Data Availability

Data are contained within the article.
